# Visualising patients' dynamics in the ICU and predicting mortality in real-time using big data

**DOI:** 10.1186/cc14579

**Published:** 2015-03-16

**Authors:** MK Komorowski, AF Faisal

**Affiliations:** 1 Imperial College London, UK

## Introduction

As informatisation of hospitals continues to spread, increasing amounts of healthcare related data are being collected, and the ICU is no exception. Large datasets are being made available to the scientific community, and offer the potential to answer clinical questions and to develop the next generation of clinical tools. A demonstration of such a tool is presented here, built using data from the Multiparameter Intelligent Monitoring in Intensive Care II (MIMIC-II) open database.

## Methods

All of the adult patients who died during their stay in the ICU were included, as well as a matched cohort of patients who survived for more than 28 days after discharge. Data regarding their vital signs, laboratory tests and demographics were collected. Using Matlab, a graphical method involving principal component analysis was developed. The expected mortality was computed using the k-nearest neighbours' method and compared with several classification algorithms (logistic regression, random forest, support vector machine, Gaussian mixture models).

## Results

A total of 6,084 patients were included in the analyses, adding up to more than 12 million data points. Using this multidimensional dataset, a 3D representation of the clusters of survivors and nonsurvivors was built, showing how their trajectories diverge through time. Patterns in the evolution of individuals or subgroups of patients can be identified using this approach. For example, the evolution of a new patient can be visualised, progressing through the clusters as his severity changes. His expected mortality can be predicted at any point in time, with an AUC ROC constantly above 0.85.

## Conclusion

Machine learning tools offer an appealing mathematical framework for modelling complex medical situations. This proof of concept demonstrates that the application of computational sciences to high-quality data such as the MIMIC-II database has the potential to lead to the development of meaningful tools which will ultimately be capable of assisting physicians in making the right decision at the right time for an individual patient. Only tight cooperation between clinicians and data scientists can help close the gap that currently separates these two worlds, for the ultimate benefit of patients.

